# Prevalence and genetic characterization of *Toxoplasma gondii* in retail meats from fresh Markets in Songkhla Province, Thailand

**DOI:** 10.1016/j.parepi.2026.e00531

**Published:** 2026-08-05

**Authors:** Siriluk Kaewmanee, Domechai Kaewnoi, Ketsarin Kamyingkird, Ruttayaporn Ngasaman

**Affiliations:** a Faculty of Veterinary Science, Prince of Songkla University, Songkhla 90110, Thailand; b Department of Parasitology, Faculty of Veterinary Medicine, Kasetsart University, Lad Yao, Chatuchak, Bangkok 10900, Thailand

**Keywords:** *Toxoplasma gondii*, GRA7 gene, Nested PCR, Food safety, Genetic heterogeneity

## Abstract

*Toxoplasma gondii* is a significant foodborne protozoon responsible for human toxoplasmosis, typically transmitted via undercooked meat, contaminated water, or contact with oocysts in cat feces. This study aimed to evaluate the prevalence and genetic diversity of *T. gondii* in retail meats within Songkhla province to assess public health risks. A total of 304 meat samples—comprising chicken (*N* = 108), pork (*N* = 107), and beef (*N* = 89)—were collected from 30 fresh markets across seven regions of Songkhla. DNA was extracted from 100 μl of meat juice and analyzed using GRA7 nested-PCR assays. Positive samples underwent sequencing and phylogenetic analysis to determine genetic lineage. The overall prevalence of *T. gondii* was 4.93% (15/304, 95% CI, 2.79–8.01%). Contamination was most frequent in poultry (10.19%), followed by pork (3.74%), while all beef samples tested negative. Geographically, the Singhanakorn-Sathing Phra (SH-ST) area exhibited the highest prevalence. Molecular analysis showed a 99.46% identity with reference GRA7 sequences (e.g., KU599267.1, KU599318.1). Notably, phylogenetic analysis revealed significant genetic heterogeneity, with some isolates (HB2, KS4) matching reference strains while others (SS1, SS4) indicated divergent lineages. These findings confirm the presence of genetically diverse *T. gondii* in the local meat supply, particularly in poultry. There is an urgent need for enhanced food safety programs, including the implementation of Good Agricultural Practices (GAP) on farms and Good Manufacturing Practices (GMP) in slaughterhouses, alongside public education regarding meat consumption habits.

## 1 Introduction

*Toxoplasma gondii* is one of the foodborne protozoa that causes human toxoplasmosis. Healthy people may show only flu like symptoms, but infections can affect the brain (toxoplasmic encephalitis), eyes (retinochoroiditis), and lungs in immuno-compromised people. The main route of transmission of human infection is caused by consumption of tissue cysts, which contain bradyzoite in undercooked meat, in particular pork, lamb, and wild game meat. Tissue cysts of *T. gondii* can persist for the life of the warm-blooded animal host (CDC, 2026). After digestion by humans, the cyst will rupture and release many bradyzoites, then grow into tachyzoites that increase infectivity (Weiss and Kim, 2000).

Approximately one-third of the global population is estimated to be chronically infected with *T. gondii*. The seroprevalences ranged from 10% to over 80%, depending on regions (Nasiru Wana et al., 2020; Bisetegn et al., 2023). In Southeast Asia, a high prevalence of human infections has been recorded in Indonesia at 58.5% (Tuda et al., 2017). Additionally, IgG and IgM antibodies were detected in Malaysia with a prevalence of 59.7% (Nasiru Wana et al., 2020). In Thailand, Toxoplasma infection is not limited to HIV patients, as evidenced by high seroprevalence rates in other groups. Research on pregnant women Southern showed an IgG positivity rate of approximately 21.6% to 22.0%, with IgM and IgG co-positivity ranging from 3.0% to 6.7% (Andiappan et al., 2014; Nissapatorn et al., 2011). Notably, certain areas such as Songkhla Province exhibit a significantly higher IgG seroprevalence of 76.2% (Chemoh et al., 2015). These figures stand in stark contrast to the general healthy population, where IgM prevalence is reported at only 3.1% (Maruyama et al., 2000).

In Thailand, the overall seroprevalence was investigated across various livestock species—including cattle, buffaloes, and goats—as well as in pets like cats and dogs, with rates ranging from 1.48% to 22.33% (Wannapong et al., 2024. Huertas-López et al., 2021, Inpankaew, 2008). Although the infected animals generally remain asymptomatic, they continue to serve as significant reservoirs for transmission. Regarding Southern regions, data on the prevalence of *T. gondii* in livestock products remains limited. Furthermore, there have been no prior studies on this pathogen in commercial meat products distributed in markets across the lower southern region. This study aimed to fill the gaps in the epidemiological data of *T. gondii* in meat produced for human consumption by doing a molecular analysis.

Primary detection methods of *T. gondii* are bioassays, cell culture, molecular and microscopic techniques, and serological methods, however, some of these do not have applicability in the food industry (Marín-García et al., 2022). The most promising alternative detection method is the polymerase chain reaction (PCR), which detects genetic material (DNA) of the tachyzoites, bradyzoites, or oocysts themselves, confirming an active presence in the sample. PCR process is fast (<1 day) and efficient diagnosis because it can detect extremely low levels of parasite DNA as well as more practical for large samples. To date, the new GRA7 nested-PCR assay gene provides high sensitivity and specificity (Costa et al., 2016). Therefore, this study aimed to apply GRA7 nested PCR for molecular detection of *T. gondii* in retail meat, aiding in reducing the time for analysis.

## 2 Materials and methods

### 2.1 Sample collection

**Fig. 1:**
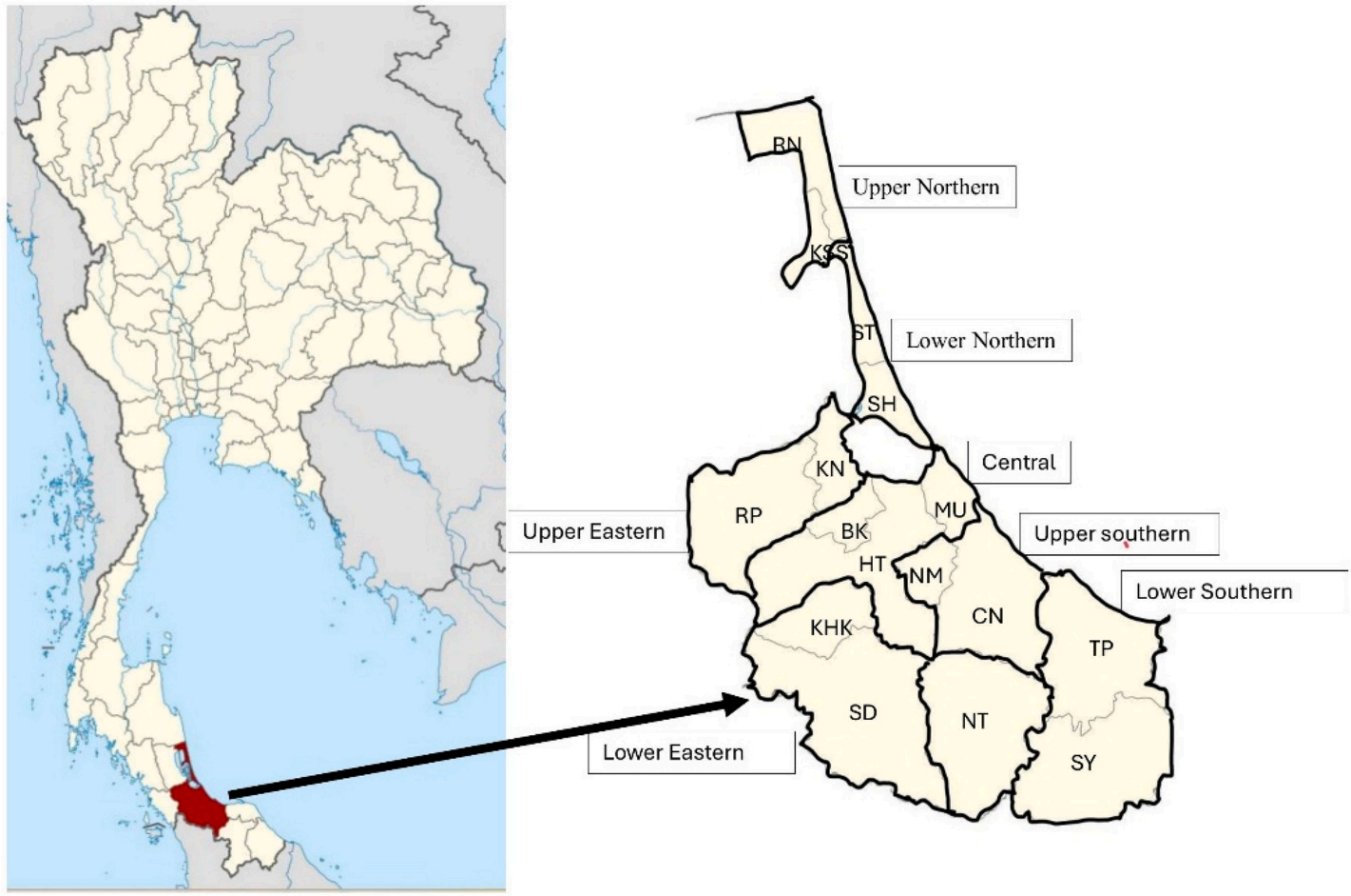
Sampling areas: fresh markets in 7 regions of Songkhla province; (1) upper northern ((3 M: *N* = 22)), (2) lower northern (4F: *N* = 43), (3) Central (8F: *N* = 89), (4) upper southern (4F: *N* = 40), (5) lower southern (3F: N = 22), (6) upper eastern (4F: *N* = 45, 7) lower eastern (4F: *N* = 43). (F; fresh marker, N; samples).

One hundred grams of each muscle meat type were randomly collected from butcher shops. Over all 304 meat samples included poultry meat (108), pork (107), and beef (89) in 30 fresh markets across 7 regions of Songkhla province were collected. The sampling areas include 30 markets from (1) upper northern (Ranot-Krasae Sin subdistricts 3 markets), (2) lower northern (Singhanakorn-Sathing Phra 4 markets), (3) Central (Hatyai-Bangkram-Maung 8 markets), (4) upper southern (Na Mom-Chana-Na Thawi 4 markets), (5) lower southern (Thepha-Saba Yoi 3 markets), (6) upper eastern (Rattaphum- Khuan Nian 4 markets) and (7) lower eastern (Khlong Hoi Khong- Sadao 4 markets). The data as shown in Fig. 1. All samples were kept at −80 degrees Celsius until used for DNA extraction.

### 2.2 DNA extraction

The freeze samples were thawed at room temperature, then 100 μl of meat juice were collected for DNA extraction. Centrifuge the juice at high speeds 14,000 rpm for 5 min to pellet. The pellet was used for DNA extraction, performed by using the GF-1 tissue DNA extraction kit (Vivantis Technologies, Selangor, Malaysia) according to the manufacturer's protocol. This process was conducted in a Biosafety Level 2 laboratory, and researchers always used personal protective equipment while working in the lab.

### 2.3 GRA7-Nested PCR

The two steps of GRA7 nested-PCR assays were performed according to Costa et al. (2016). Briefly, a total volume of 12 μl reactions containing 2 μl of template DNA, 0.25 μM of each primer, 5 μl of Taq polymerase master mix (KAPA®, Japan) and 4.5 μl of ultra-pure water was subjected for PCR amplification. The first step PCRs were performed using the primer pairs GRA7FE/GRA7RE and nested-PCRs using GRA7FI/GRA7RI (Table 1) which the same condition. The PCR cycle consisted of an initial denaturation step at 94 °C for 2 min, followed by 35 cycles of 40 s at 94 °C for denaturation, 40 s at 59 °C for annealing and 1 min at 63 °C for extension, with a final extension at 63 °C for 3 min. PCR products were resolved in a 2% agarose gel stained with ethidium bromide to get the size of 322 and 222 base pairs for the 1st and 2nd step, respectively.

**Table 1:** Primers for nested-polymerase chain reaction targets GRA-7 gene of *T. gondii* (Costa et al., 2016).

Primer names	Primer sequence (5′-3′)	Product size (bp)
GRA7FE	CAAGCACCCGTTGACAGTCT	322
GRA7RE	ACGATGCACCCATACCAACAG	
GRA7FI	CACCAGCATGGATAAGGCATC	222
GRA7RI	GCGAGCTTCTTCAGCAAGTCT	

### 2.4 Sequencing and phylogenetic analysis

The positive PCR products were selected; 4 poultry meat (HB2, KS4, SS1, SS4) and 1 pork meat (KS2) were sent for nucleotide sequencing using the Sanger method. Nucleotide sequences were analyzed using Basic Local Alignment Search Tool (BLAST) on the National Center for Biotechnology Information (NCBI) platform (https://blast.ncbi.nlm.nih.gov). Multiple alignments were performed using Clustal W, and phylogenetic trees were constructed using MEGA (version 12) software. The evolutionary history was inferred using the Neighbor-Joining method and the Tamura-Nei model (Tamura and Nei, 1993). To evaluate the reliability of the tree topology, bootstrap analysis was performed with 1000 replicates. The genetic distance scale was set at 0.01 substitutions per site. The reference sequences were selected from standard databases NCBI, focusing on type strains.

### 2.5 Statistical analysis

The proportion of positive values was calculated, with statistical significance defined as *P* < 0.05 at a 95% confidence interval. Inter-group comparisons were performed using the Chi-square test. Statistical analyses were conducted via online tools (https://sample-size.net and https://www.socscistatistics.com).

## 3 Results

**Fig. 2:**
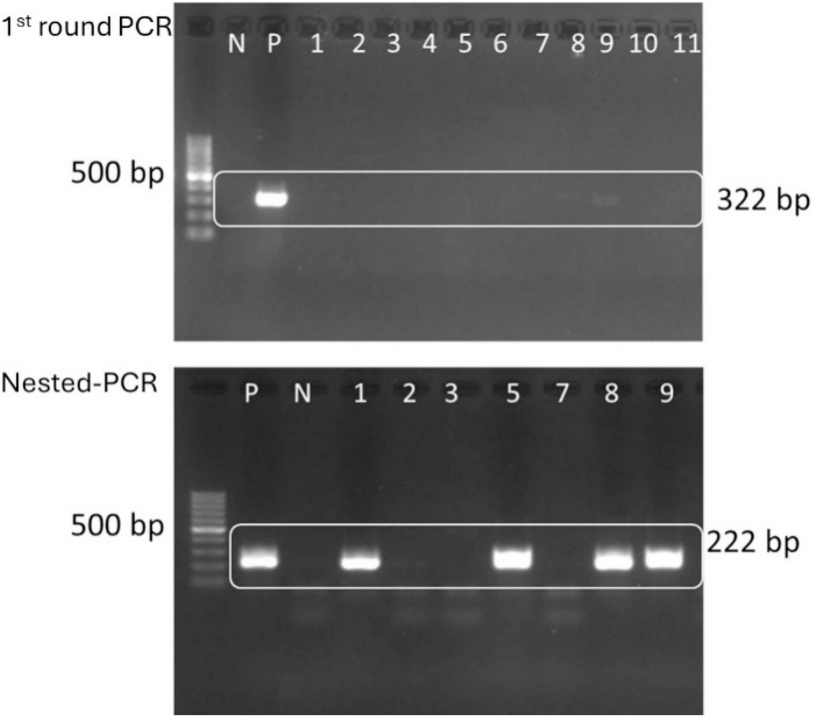
Gel electrophoresis of PCR product from 1st round and nested PCR at 322 and 222 base pairs.

Application of GRA7 nested PCR, the first step, PCR targeted the external conserved region of GRA7 gene at 322 bp sequence. The second step amplified a specific fragment of 222 bp (Fig. 2). 15 out of 304 samples were positive by GRA7 nested PCR (4.93%, *95% CI: 2.79–8.01*). Among positive samples, 11 were poultry meat (11/108: 10.19%*)* and 4 were pork meat (4/107: 3.74%) but not found in beef. When comparing the groups, no significant differences in positive results were found between chicken and pork, or between pork and beef. However, a statistically significant difference was observed between chicken and beef (*p-value* = 0.0019).

The region that showed the highest overall positive results was SH-ST (11.13%) followed by NM-JN-TW (5.00%), RN-KSS (4.76%), KHK-SD (4.65%), RP-KN (4.44%), HT-BK-MU (3.37%). Positive poultry meat mostly found from fresh markets around SH-ST (18.75%) followed by NM-JN-TW (13.33%), RP-KN (13.33%), RN-KSS (9.09%), HT-BK-MU (7.14%), KHK-SD (6.67%). Positive pork meats were from the fresh market around SH-ST (14.29%), KHK-SD (6.67%) and HT-BK-MU (3.33%) (Table 2).

**Table 2:** Positive results of *T. gondii* by GRA nested PCR in 3 types of meat (beef, poultry and pork) from fresh markets (30) across 7 regions of Songkhla province.

Sampling areas	No. fresh market	Poultry			Pork			Beef		Pos/Total samples	Total (%, (95%CI))
		No. Sample	No. Positive	% (95%CI)	No. Sample	No. Positive	% (95%CI)	No. Sample	No. Positive		
Upper Northern	3	11	1	9.09 *(0.23–41.28)*	10	0	0	1	0	1/22	4.76 (*0.12–23.82*)
Lower Northern	4	16	3	18.75 *(4.05–45.65)*	14	2	14.29 *(1.78–42.81)*	13	0	5/43	11.63 (*3.89–25.08*)
Central	8	28	2	7.14 *(0.9–23.5)*	30	1	3.33 *(0.08–17.22)*	31	0	2/89	3.37 (*0.7–9.54*)
Upper Southern	4	15	2	13.33 *(1.66–40.46)*	12	0	0	13	0	2/40	5.00 (*0.6–16.92*)
Lower Southern	3	8	0	0	9	0	0	5	0	0/22	0
Upper Eastern	4	15	2	13.33 *(1.66–40.46)*	17	0	0	13	0	2/45	4.44 (*0.54–15.15*)
Lower Eastern	4	15	1	6.67 (*0.17–31.95)*	15	1	6.67 (*0.17–31.95)*	13	0	2/43	4.65 (*0.57–15.85*)
Total	30	108	11^A^	10.19 *(5.19–17.49)*	107	4^B^	3.74 (*1.03–9.30*)	89	0^C^	14/304	4.93 (*2.79–8.01*)
*p-values*			0.0635			0.0650			0.0019		

A Between poultry and pork.

B Between pork and beef.

C Between poultry and beef.

The BLAST analysis revealed that the sequence obtained from the poultry samples (HB2, KS4, SS1, SS4) exhibited 99.46% identity with the *T. gondii* dense granule protein (GRA7) gene. The high query coverage (94%) and a significant *E*-value (2e-87) are similar with TgH18013, GUY-2004-LAB, and TgCatTr_Izmir02. The positive pork samples (KS2) showed a high degree of genetic similarity to *T. gondii* dense granule protein (GRA7) gene 99.46% (e.g., Accession No. KU599267.1) with a significant *E*-value of 2e-87 and high query coverage (93–94%). Interestingly, one positive sample form poultry (SS2) was identified as *Acinetobacter johnsonii* strain FDAARGOS_1092 (Accession: CP068206.1) (97.63%) with a significant E-value of 2e-73.

**Fig. 3:**
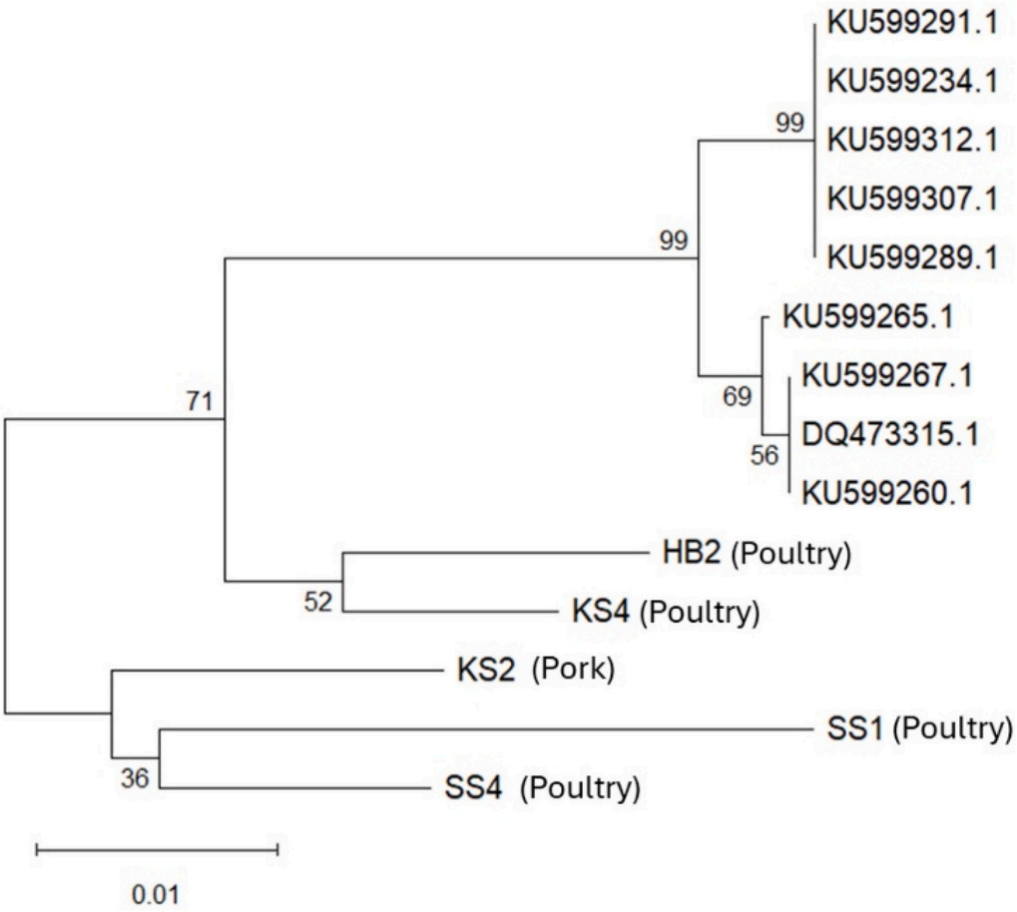
Phylogenetic tree of *Toxoplasma gondii* isolates based on GRA7 gene sequences. The tree was constructed using the Neighbor-Joining (NJ) method with 1000 bootstrap replicates. Numbers at the nodes indicate bootstrap values (%) above 50. The scale bar represents 0.01 substitutions per nucleotide site. Isolates from this study are designated by their sample IDs (e.g., HB2, KS4, KS2, SS1, and SS4) along with their respective meat sources (Poultry, Pork). Reference sequences were obtained from GenBank and are indicated by their accession numbers.

The analysis reveals genetic heterogeneity among the poultry isolates. While HB2 and KS4 are phylogenetically aligned with established reference strains, the SS1 and SS4 isolates represent a divergent lineage. The distinct positioning of the KS2 (Pork) isolate further highlights the genetic diversity present across different host species in this dataset (Fig. 3).

## 4 Discussion

This study determined the overall prevalence of *T. gondii* in retail meats of Songkhla province 4.93%. This suggested a persistent environmental presence of *T. gondii* oocysts or tissue cysts in the livestock supply chain. Positive finding in poultry meat was higher than pork but did not find in beef. The higher prevalence in poultry (10.19%) compared to pork (3.74%) is noteworthy. This may be attributed to poultry farming practices, where birds (especially free-range) have frequent contact with soil contaminated by felid feces. Previously, the overall *Toxoplasma* seroprevalence in free-range chickens in Thailand was 33.0% and 17.7% by IFAT and MAT, respectively (Udonsom et al., 2017). However, there have been no reports regarding poultry meat products in Thailand. In Greece, the overall *T. gondii* seroprevalence in backyard chickens was 9.5%, while those in organic and free-range layer farms were 41.2% and 70%, respectively (Andreopoulou et al., 2023). The prevalence rate of *T. gondii* in chickens was 72.9% by IHA and 83.1% by PCR in Southern Syria (Mahmood and Al Nahhas, 2025). In Taipei, it was determined that the prevalence of *T. gondii* DNA was 8% in pork, 2% in pig liver, 4% in mutton, 4% in chicken flesh, 2% in chicken heart, and 5% in imported beef (Fuh et al., 2013).

*T. gondii* is a common infection within the pig farming industry. In Northern Thailand, the seroprevalence of *T. gondii* among slaughtered pigs was reported at 7.8% (Sutjarit et al., 2009). In China, seropositivity rates reached up to 70% in specific areas and farms (Pan et al., 2017). Furthermore, the overall seroprevalence in slaughtered pigs in Kiambu, Kenya, and Egypt was 34.53% (Chepyatich et al., 2023) and 45.8% (Fereig et al., 2023), respectively. At abattoirs in Gifu Prefecture, Japan, antibodies to *T. gondii* were detected in 7.3% of cattle and 5.2% of pigs (Matsuo et al., 2014). Additionally, this research indicated the prevalence of *T. gondii* in raw pork (3.74%) was higher than in minced pork (1.38%) sourced from morning markets in Bangkok, Thailand, (Sutthikornchai et al., 2013).

Although no positive samples were detected in the beef samples in this study, the prevalence in Western regions of Thailand has been reported seroprevalence to range from 15.2% - 25.7% by ELISA and IFAT technique, respectively (Udonsom et al., 2018; Wiengcharoen et al., 2012). Interestingly, the first report of molecular detection *T. gondii* in blood samples of goat from Northern Thailand was 8.52% (Koonyosying et al., 2025).

The Singhanakorn-Sathing Phra fresh market emerged as a high-risk area for both poultry (18.75%) and pork (14.29%). This concentration suggests either a localized high environmental parasite load or shared supply chains that are particularly vulnerable to contamination. The findings may be attributed to substandard agricultural practices and non-standardized slaughtering processes in both poultry and pig production. Furthermore, since wooden cutting boards were commonly used by retail sellers, cross-contamination could occur due to inadequate cleaning and disinfection procedures. These results indicate a potential risk of *T. gondii* infection from consuming undercooked pork. Given that contamination can occur at any stage from pig farms to meat shops, a robust pork traceability system should be implemented.

The phylogenetic analysis revealed significant genetic diversity among the isolates collected from different hosts and locations. The high bootstrap support (99%) at the node of the primary cluster suggests a stable evolutionary lineage for the KU599… reference group. The inclusion of HB2 (Poultry) and KS4 (Poultry) within this clade indicates that these isolates share a recent common ancestor with the reference strains, suggesting a potentially widespread genotype in the poultry population. In contrast, the SS1 and SS4 isolates formed a separate, more divergent branch. Although the bootstrap support for the SS-clade was relatively low (36%), their clear separation from the main reference cluster implies the presence of a distinct sub-lineage circulating in the same region. Furthermore, the KS2 (Pork) isolate occupied an intermediate position between the SS-group and the main cluster, highlighting that host-specific factors (Pork vs. Poultry) may contribute to the observed genetic variations. The branch lengths observed for the SS (1 and 4) and KS2 isolates suggest a higher rate of mutation or a longer period of independent evolution compared to the more conserved HB2 and KS4 strains.

Interestingly, one positive sample was sequenced as bacteria *Acinetobacter johnsonii.* This might be because of contamination as well as non-specific amplification. *A. johnsonii* bacterium is an opportunistic Gram-negative, non-fermenting, coccobacilli found in the environment, on human skin and animal skin (including fish and cattle) (Al Atrouni et al., 2016). Previously it was considered as a low-virulence environmental organism. It has occasionally been reported as emerging pathogens cause meningitis (Gutiérrez-Gaitán et al., 2022). The presence of *A. johnsonii* in the poultry meat may be attributed to poor water quality in the slaughterhouse, as well as cross-contamination during the defeathering and evisceration stages, or insufficient disinfection of slaughtering tools. Moreover, this may reflect sanitary deficiencies in the post-harvest process at the fresh market. Furthermore, it underscores the critical necessity of stringent temperature control during transportation and retail displays to prevent the proliferation of psychotropic bacteria. Although *A. johnsonii* is not considered as highly virulent an opportunistic pathogen like *A. baumannii*, some research has identified its potential to act as a ‘vector’ for the dissemination of antimicrobial resistance (AMR) genes (Montaña et al., 2016; Tobin et al., 2024).

A limitation of this study is that PCR detects parasite DNA rather than providing evidence of parasite viability. The GRA7 primer used for PCR technique in this research was found to be nonspecific for the detection of *T. gondii* because after sequence one positive sample, it was detected as bacteria.

## 5 Conclusion

This research provided robust confirmation of the presence of *T. gondii* in the examined chicken and pork samples. These findings underscore the genetic diversity of *T. gondii* across different hosts and highlight a significant public health risk. To mitigate this, public education regarding meat preparation is essential. Furthermore, food safety programs—specifically Good Agricultural Practices (GAP) at the farm level and Good Manufacturing Practices (GMP) in slaughterhouses—must be strictly implemented.

## CRediT authorship contribution statement

Siriluk Kaewmanee: Conceptualization, Methodology, Investigation, Writing- Original draft preparation Domechai Kaewnoi: Investigation, Data curation. Ketsarin Kamyingkird: Visualization, Supervision. Ruttayaporn Ngasaman: Software, Validation, Writing- Reviewing and Editing.

## Ethics statement

The authors declared that they have no financial or personal relationships that may have inappropriately influenced them in writing this article.

## Funding

This research was financially supported by the 10.13039/501100004508Faculty of Veterinary Science, Prince of Songkla University for the funding of Academic Year 2024 (Grant no. VET6704093S).

## Declaration of competing interest

The authors declare that they have no competing financial interests or personal relationships that could have appeared to influence the work reported in this paper.

## References

[bb0005] Al Atrouni A., Joly-Guillou M.-L., Hamze M., Kempf M. (2016). Reservoirs of non-baumannii *Acinetobacter* species [mini review]. Front. Microbiol..

[bb0010] Andiappan H., Nissapatorn V., Sawangjaroen N., Chemoh W., Lau Y.L., Kumar T., Onichandran S., Suwanrath C., Chandeying V. (2014). Toxoplasma infection in pregnant women: a current status in Songklanagarind hospital, southern Thailand. Parasit. Vectors.

[bb0015] Andreopoulou M., Schares G., Koethe M. (2023). Prevalence and molecular characterization of *Toxoplasma gondii* in different types of poultry in Greece, associated risk factors and co-existence with *Eimeria* spp. Parasitol. Res..

[bb0020] Bisetegn H., Debash H., Ebrahim H., Mahmood N., Gedefie A., Tilahun M., Alemayehu E., Mohammed O., Feleke D.G. (2023). Global seroprevalence of *toxoplasma gondii* infection among patients with mental and neurological disorders: A systematic review and meta-analysis. Health Sci. Rep..

[bb0025] CDC (2026). Toxoplasmosis. https://www.cdc.gov/dpdx/toxoplasmosis/index.html.

[bb0030] Chemoh W., Sawangjaroen N., Siripaitoon P., Andiappan H., Hortiwakul T., Sermwittayawong N., Charoenmak B., Nissapatorn V. (2015). *Toxoplasma gondii* - prevalence and risk factors in HIV-infected patients from Songklanagarind hospital, Southern Thailand. Front. Microbiol..

[bb0035] Chepyatich D., Sentamu D.N., Bor N., Onono J., Gathura P.B., Akoko J.M., Thomas L.F. (2023). Seroprevalence of *Toxoplasma gondii* in slaughtered pigs in Kiambu, Kenya. Zoonotic Dis..

[bb0040] Costa M.E.S.M., Oliveira C.B.S., Andrade J.M.d.A., Medeiros T.A., Neto V.F.A., Lanza D.C.F. (2016). An alternative nested-PCR assay for the detection of *Toxoplasma gondii* strains based on GRA7 gene sequences. Acta Trop..

[bb0045] Fereig R.M., El-Alfy E.S., Abdelbaky H.H., Abdel-Hamid N.H., Mazeed A.M., Menshawy A.M.S., Kelany M.A., El-Diasty M., Alawfi B.S., Frey C.F. (2023). Seroprevalence of *Toxoplasma gondii*, *Neospora caninum* and *Trichinella* spp. in pigs from Cairo, Egypt. Vet. Sci..

[bb0050] Fuh Y.-B.T., Liao A.T., Pong Y.-M.T., Tung M.-C.T., Fei C.-Y.T., Lin D.-S. (2013). Survey of *Toxoplasma gondii* in Taipei: livestock meats, internal organs, cat and dog sera. Thai J. Vet. Med..

[bb0055] Gutiérrez-Gaitán M.P., Montoya-Moncada A.D., Suescún-Vargas J.M., Pinzón-Salamanca J.Y., Aguirre-Borrero B.L. (2022). Emerging species in pediatrics: a case of *Acinetobacter johnsonii* meningitis. Bol. Med. Hosp. Infant. Mex..

[bb0060] Huertas-López A., Sukhumavasi W., Álvarez-García G., Martínez-Subiela S., Cano-Terriza D., Almería S., Martínez-Carrasco C. (2021). Seroprevalence of *Toxoplasma gondii* in outdoor dogs and cats in Bangkok, Thailand. Parasitology.

[bb0065] Inpankaew T. (2008). Seroprevalence of *Toxoplasma gondii* infection in dairy cows in Northeastern Thailand. Southeast Asian J. Trop. Med. Public Health.

[bb0070] Koonyosying P., Muenthaisong A., Sangkakam K., Nontasaya K., Rittipornlertrak A., Nambooppha B., Apinda N., Maneekesorn S., Sthitmatee N. (2025). Molecular detection of genetic material of *Toxoplasma gondii* in goat blood samples from northern Thailand. Vet. Sci..

[bb0075] Mahmood D., Al Nahhas S. (2025). Serological and molecular detection of *Toxoplasma gondii* in chickens in southern Syria. Food Waterborne Parasitol..

[bb0080] Marín-García P.-J., Planas N., Llobat L. (2022). *Toxoplasma gondii* in foods: prevalence, control, and safety. Foods.

[bb0085] Maruyama S., Boonmar S., Morita Y., Sakai T., Tanaka S., Yamaguchi F., Kabeya H., Katsube Y. (2000). Seroprevalence of *Bartonella henselae* and *Toxoplasma gondii* among healthy individuals in Thailand. J. Vet. Med. Sci..

[bb0090] Matsuo K., Kamai R., Uetsu H., Goto H., Takashima Y., Nagamune K. (2014). Seroprevalence of *Toxoplasma gondii* infection in cattle, horses, pigs and chickens in Japan. Parasitol. Int..

[bb0095] Montaña S., Schramm S.T., Traglia G.M., Chiem K., Parmeciano Di Noto G., Almuzara M., Barberis C., Vay C., Quiroga C., Tolmasky M.E., Iriarte A., Ramírez M.S. (2016). The genetic analysis of an *Acinetobacter johnsonii* clinical strain evidenced the presence of horizontal genetic transfer. PLoS One.

[bb0100] Nasiru Wana M., Mohd Moklas M.A., Watanabe M., Nordin N., Zasmy Unyah N., Alhassan Abdullahi S., Ahmad Issa Alapid A., Mustapha T., Basir R., Abd. Majid R. (2020). A review on the prevalence of *Toxoplasma gondii* in humans and animals reported in Malaysia from 2008–2018. Int. J. Environ. Res. Public Health.

[bb0105] Nissapatorn V., Suwanrath C., Sawangjaroen N., Ling L.Y., Chandeying V. (2011). Toxoplasmosis-serological evidence and associated risk factors among pregnant women in southern Thailand. Am. J. Trop. Med. Hyg..

[bib152] Pan M., Lyu C., Zhao J., Shen B. (2017). Sixty years (1957-2017) of research on toxoplasmosis in China-an overview. Front. Microbiol..

[bb0110] Sutjarit S., Inthong N., Areesrisom P., Mongkut J., Rerkamnuaychoke W., Pinyopanuwat N., Kengradomgit C., Jittapalapong S. (2009). Proceedings of the 47th KasetsarvUniversity Academic Meeting.

[bb0115] Sutthikornchai C., Mahitikorn A., Sukthana Y. (2013). Quantitative PCR detection of *Toxoplasma gondii* in minced pork from selected morning markets in Bangkok, Thailand. J. Trop. Med. Parasitol..

[bib151] Tamura K., Nei M. (1993). Estimation of the number of nucleotide substitutions in the control region of mitochondrial DNA in humans and chimpanzees. Mol. Biol. Evol..

[bb0120] Tobin L., Jarocki V., Kenyon J., Drigo B., Donner E., Djordjevic S., Hamidian M. (2024). Genomic analysis of diverse environmental Acinetobacter isolates identifies plasmids, antibiotic resistance genes, and capsular polysaccharides shared with clinical strains. Appl. Environ. Microbiol..

[bb0125] Tuda J., Adiani S., Ichikawa-Seki M., Umeda K., Nishikawa Y. (2017). Seroprevalence of *Toxoplasma gondii* in humans and pigs in North Sulawesi, Indonesia. Parasitol. Int..

[bb0130] Udonsom R., Chaichan P., Mahittikorn A., Vignoles P., Mercier A., Aroussi A., Dardé M.-L. (2017). *Toxoplasma* serostatus in Thai free- range chickens: prevalence and two diagnostic methods. Open J. Trop. Med..

[bb0135] Udonsom R., Sukthana Y., Nishikawa Y., Fereig R.M., Jirapattharasate C. (2018). Current situation of *Neospora caninum* and *Toxoplasma gondii* infection among beef cattle in Kanchanaburi, Ratchaburi and Nakhon Patom provinces, Thailand. Thai J. Vet. Med..

[bb0140] Wannapong N., Lertwatcharasarakul P., Rukkwamsuk T. (2024). Prevalence and Risk Factors of *Toxoplasma Gondii* Infection in Dairy Cattle from the Western Region of Thailand. Prévalence et Facteurs de Risque de L’infection à Toxoplasma Gondii Chez les Bovins Laitiers de la région Occidentale de la Thaïlande. Parasite.

[bb0145] Weiss L.M., Kim K. (2000). The development and biology of bradyzoites of *Toxoplasma gondii*. Front. Biosci..

[bb0150] Wiengcharoen J., Nakthong C., Mitchaothai J., Udonsom R., Sukthana Y. (2012). Toxoplasmosis and neosporosis among beef cattle slaughtered for food in Western Thailand. Southeast Asian J. Trop. Med. Public Health.

